# Sol-Gel Synthesis of Ceria-Zirconia-Based High-Entropy Oxides as High-Promotion Catalysts for the Synthesis of 1,2-Diketones from Aldehyde

**DOI:** 10.3390/molecules26206115

**Published:** 2021-10-10

**Authors:** Dalibor Tatar, Jelena Kojčinović, Berislav Marković, Aleksandar Széchenyi, Aleksandar Miletić, Sándor Balázs Nagy, Szilveszter Ziegenheim, Imre Szenti, Andras Sapi, Ákos Kukovecz, Kristijan Dinjar, Yushu Tang, David Stenzel, Gábor Varga, Igor Djerdj

**Affiliations:** 1 Department of Chemistry, Josip Juraj Strossmayer University of Osijek, Cara Hadrijana 8/A, HR-31000 Osijek, Croatia; dtatar@kemija.unios.hr (D.T.); jelena.bijelic@kemija.unios.hr (J.K.); bmarkovi@kemija.unios.hr (B.M.); szealex@kemija.unios.hr (A.S.); 2 Faculty of Technical Sciences, University of Novi Sad, Trg Dositeja Obradovića 6, SRB-21000 Novi Sad, Serbia; aleksandarvmiletic@gmail.com; 3 Department of Organic Chemistry, University of Szeged, Dóm tér 8., H-6720 Szeged, Hungary; sandorb@chem.u-szeged.hu (S.B.N.); ziegenheimsz@chem.u-szeged.hu (S.Z.); 4 Department of Applied and Environmental Chemistry, University of Szeged, Rerrich Béla Sq. 1., H-6720 Szeged, Hungary; szentiimre@gmail.com (I.S.); sapia@chem.u-szeged.hu (A.S.); kakos@chem.u-szeged.hu (Á.K.); 5 Department of Otorhinolaryngology and Maxillofacial Surgery, Faculty of Medicine, Josip Juraj Strossmayer University of Osijek, Cara Hadrijana 10/E, HR-31000 Osijek, Croatia; kristijan.dinjar@kbco.hr; 6 Karlsruhe Institute of Technology (KIT), Institute of Nanotechnology, Hermann-von-Helmholtz-Platz 1, DE-76344 Eggenstein-Leopoldshafen, Germany; yushu.tang@kit.edu (Y.T.); david.stenzel@kit.edu (D.S.); 7 Department of Physical Chemistry and Materials Science, University of Szeged, Rerrich Béla Sq. 1., H-6720 Szeged, Hungary

**Keywords:** 1,2-diketone-selective conversion of aldehydes, ceria, ceria–zirconia-based multimetallic oxides, high-entropy oxides (HEOs), Lewis acid-promoted direct synthesis of 1,2-diketone, pinacol-type oxidative coupling of aldehydes, sol-gel synthesis

## Abstract

Efficient Lewis-acid-catalyzed direct conversion of aldehydes to 1,2-diketones in the liquid phase was enabled by using newly designed and developed ceria–zirconia-based high-entropy oxides (HEOs) as the actual catalysts. The synergistic effect of various cations incorporated in the same oxide structure (framework) was partially responsible for the efficiency of multicationic materials compared to the corresponding single-cation oxide forms. Furthermore, a clear, linear relationship between the Lewis acidity and the catalytic activity of the HEOs was observed. Due to the developed strategy, exclusively diketone-selective, recyclable, versatile heterogeneous catalytic transformation of aldehydes can be realized under mild reaction conditions.

## 1. Introduction

With the rapid development of the industry, one of the main objectives in materials science: finding new, advanced materials, has become a priority. One of the newest classes of those materials, which has attracted much scientific attention, is high-entropy oxides (HEOs). They were first reported as entropy-stabilized oxides by Rost et al. [1]. These materials represent clear evidence that five or more different cations can be incorporated into a single-phase oxide system. However, to obtain such a system, several conditions need to be met. General principles, when choosing appropriate constituents, rely on the consideration of ionic radii, the oxidation state, and the coordination number of the cations that will occupy a single-cation sublattice, while the anion sublattice is only occupied by oxygen [2,3,4,5,6,7,8]. Since their discovery, several further studies have been reported with a focus on the properties of these materials [9,10] and the development of new HEOs [2,3,5,7,11,12].

Ceria-based multicomponent oxides are some of the most interesting groups of HEOs, in the application context. Such materials are top quality for application in solid-oxide fuel cells and solid-oxide electrolysis cells (SOFCs and SOECs), which are some of the most promising technologies that include chemical to electrical and electrical to chemical energy conversion applications [13]. Despite the appearance of ever-newer applications of ceria-based HEOs, there is no example of applying them as actual Lewis acid catalysts, even though each metal oxide on its own is well known as an efficient catalyst. This is striking because uncountable studies have already focused on the organic catalytic transformations promoted by cerium-[14], zirconium-[15], or lanthanum-based oxides [16] and their composites. Zirconium-based oxides seem to be the most attractive water-resistant Lewis acids to catalyze redox (MPVO) or coupling reactions under exceedingly mild reaction conditions [17].

Because of their antitumor and photochemical activities, among others, much attention has been given to the synthesis of 1,2 diketones and their derivatives for a long time [18,19,20,21,22]. In this context, several synthesis strategies have been designed and developed along with methodologies under catalytic conditions [23,24,25]. Although notable progress has been attained with precious noble metal catalysts [26], considerable advances have been obtained by using Lewis acids, which have more profitable qualities than noble metals [27,28]. Moreover, these catalysts have proven to be well fitted to other catalytic views, such as low toxicity and significant functional group tolerance. However, to perform the well-optimized reaction sequence, which consists of a Sonogashira-type decarboxylative coupling and oxidation, the application of arylboronic acids or aryl iodides with alkynyl carboxylic acids as raw materials is essential, substantially reducing the eco-friendly feature of these systems [26,27,28]. There is a less familiar fact that an oxidative pinacol-type coupling should be also sufficient to produce diketones that can be also promoted by Lewis acids [29]. However, maximum efficiency can be achieved by adding organic additives in these cases. Notably, to the best of our knowledge, there are no heterogeneous counterparts of these Lewis acid catalysts that were able to provide such a versatile catalytic behavior as homogeneous ones. All the reported examples to perform efficient synthesis of 1,2-diketones require the precious noble metal palladium [30].

The above trends have motivated research to explore the (Lewis) catalytic ability of HEOs to promote organic transformations in the liquid phase. To present their capacity, the liquid phase synthesis of 1,2-diketones from aldehydes was chosen as a test reaction. Notable advances of our approach, including (I) unusual recyclable Lewis-acid-catalyzed reaction, (II) an effective 1,2-diketone yield with sufficient scope, and (III) mild reaction conditions were to be enabled by the application of HEOs as the actual catalysts. Furthermore, a clear relationship between the catalytic activity and the Lewis acidity of HEOs was able to be identified.

In this research study, we synthesized and characterized four single-phase ceria–zirconia rare-earth high-entropy oxides in the cubic phase, as shown in Table 1. The involved cations were selected because of their similar ionic radii, similar oxidation state, and the same coordination number. It has been previously reported that the method of doping ceria with rare-earth cations has been widely used to improve the activity, selectivity, and thermal stability of ceria catalysts and to increase oxygen vacancy concentrations [31,32,33].

## 2. Results and Discussion

### 2.1. Structural Characterization of the HEOs

**Figure 1:**
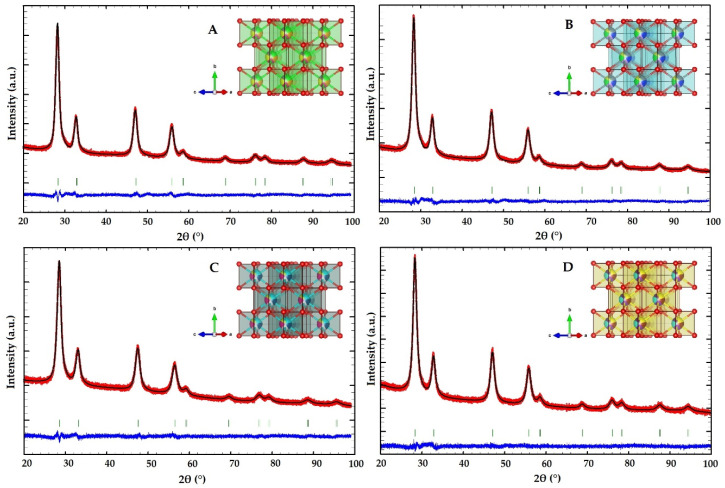
Rietveld plot of the investigated compounds: (**A**) CZLEY; (**B**) CZLPY; (**C**) CZEYG; (**D**) CZLPG. Along with Bragg reflections, the observed (red), calculated (black), and difference (blue) plots are shown for the fit of the PXRD pattern. In the insets of each figure, the fluorite-type crystal structure of the corresponding HEO is visualized.

To investigate the crystal structure, along with the structural parameters and phase purity of the synthesized materials, powder X-ray diffraction was performed. Structural details were provided by Rietveld refinement and are shown in Table S1. All Rietveld output plots reveal the phase purities of the synthesized catalysts and are presented in Figure 1. The investigated HEOs crystallized in the fluorite structure with the cubic *Fm-*3*m* space group, with similar densities and cell volumes. The lattice parameters are similar, equal to *a* = 5.4558(2) Å for CZLEY, *a* = 5.4517(2) Å for CZLPY, *a* = 5.4583(2) Å for CZEYG, and *a* = 5.4601(2) Å for CZLPG, but differ from the pure CeO_2_ extracted by Sarkar et al., where *a* = 5.4073(1) Å [34]. This shows that, the addition of cations, with the same coordination number and similar ionic radii (0.97 Å for Ce^4+^, 0.84 Å for Zr^4+^, 1.16 Å for La^3+^, 1.126/0.96 Å for Pr^3+/4+^, 1.066 Å for Eu^3+^, 1.019 Å for Y^3+^, and 1.053 Å for Gd^3+^) into a single-crystal lattice, originates the expansion of the lattice. Additionally, one can notice that the lattice parameter values depend solely on the values of the ionic radii, since they remain similar. The average crystallite size is 6 nm for CZLEY, CZLPY, and CZLPG and 5 nm for CZEYG, as calculated by the line broadening method. Microstrain values (Table S1) vary for different compositions of the HEOs, which shows that CZLPY has the highest, while CZEYG has the lowest degree of crystal lattice order. The visualized structure shows that there are five different cations on the same crystallographic position, which occupy a highly symmetric, fluorite structure of CeO_2_ and are coordinated with eight O atoms on the edges, as shown in the insets of Figure 1A–D.

**Figure 2:**
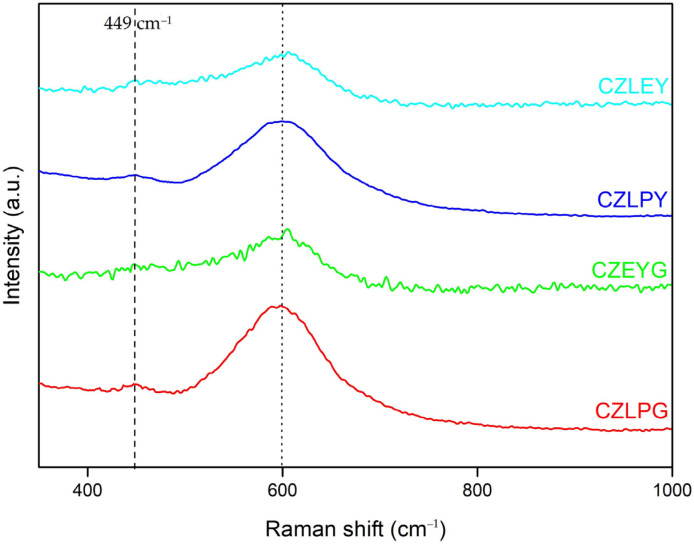
Raman spectra of the synthesized HEOs.

For advanced structural characterization of the synthesized catalysts, Raman spectroscopy was performed. Figure 2 shows the Raman spectra (*λ* = 532 nm) for the different synthesized catalysts. A single sharp band at 464 cm^−1^ is typical for CeO_2_ with the fluorite structure [35] and represents the *F_2g_* symmetric vibration mode of the eight-fold Ce-O bonds. The intensity of the sharp *F_2g_* band decreases with the addition of more elements into a ceria crystal lattice [34]. Due to the five cations incorporated into a single-crystal lattice of CeO_2_, there is an *F_2g_* band shift of ~15 cm^−1^, which is related to the expansion of the crystal lattice, the bond lengths, and the formation of oxygen defects [34,36,37]. Additional bands, which for which the synthesized HEOs differ from pure CeO_2_, are visible at 597 cm^−1^ for CZLPG, 605 cm^−1^ for CZEYG, 600 cm^−1^ for CZLPY, and 603 cm^−1^ for CZLEY, and they are related to oxygen defects [38,39]. Quantitative information about the amount of oxygen vacancies cannot be provided from Raman spectra. However, to compare the number of oxygen defects presented in the synthesized HEOs, relative oxygen vacancy concentrations were calculated as the ratio of the integral intensities of additional, defect bands (*II_D_*) and the integral intensities of the corresponding *F_2g_* bands (*II_F2g_*) [34,40]. The relative oxygen vacancy concentrations are shown in Table S2. Although oxygen vacancy concentrations are responsible for the catalytic activity of a material, it is hard to elucidate the catalytic activity only from the Raman data. Therefore, a few more parameters need to be considered, such as acidity, crystallite size, surface area, etc., as explained further in the text.

**Figure 3:**
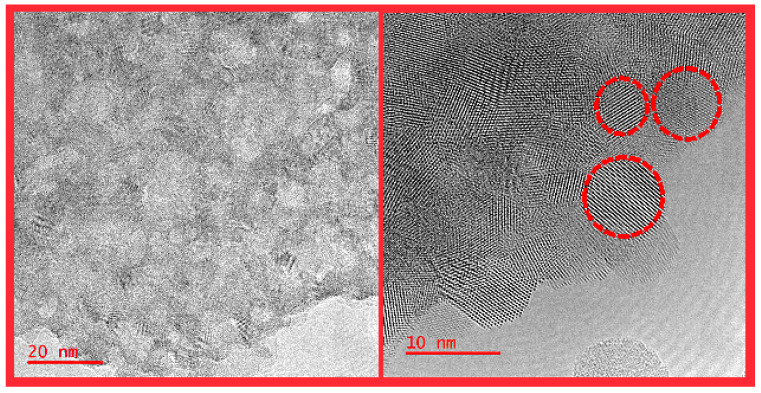
TEM/HRTEM images of the CZLPY powder sample at different magnifications.

To investigate the morphology, SEM and STEM-EDX measurements were performed. Figure S1 reveals the surface morphology of the prepared catalysts. To visually inspect crystallites in the powder samples, the CZLPY compound was additionally inspected by high-resolution transmission electron microscopy (HRTEM). Figure 3 confirms that this compound consists of agglomerated crystallites with values slightly below 10 nm, which corroborates the microstructural results obtained from the Rietveld refinement of powder XRD patterns.

Qualitative EDX maps (Figure S2) reveal the uniform distribution of the involved elements inside the powder sample. Since La-Ce-Pr and Y-Zr have similar molar mass values, it is difficult to precisely quantify the atomic content of the sample. This explains the slight deviation in the nominal and experimental values (Table S3). The EDX spectrum (Figure S3) also shows the presence of the Au, C (from the TEM grid), and Cu (TEM holder) signals. Additionally, the empirical formula of CZLPY was calculated from atomic fractions obtained from quantitative EDX. The oxygen content was further normalized to the value of two [41] giving the formula of Ce_0.18_Zr_0.14_La_0.2_Pr_0.2_Y_0.18_O_2_, which is very close to the actual formula of Ce_0.2_Zr_0.2_La_0.2_Pr_0.2_Y_0.2_O_2_. Since quantitative EDX analysis is of limited accuracy and reliability, ICP-MS elemental analysis was used for the quantification of the metal cations for all four compounds. The obtained elemental compositions (Table S4) are expressed as measured mass percent fractions accompanied by theoretical (nominal) elemental compositions. Taking into account the error bars for the measured mass percent fractions, one can rationalize that the elemental compositions of the studied HEOs correspond to their aimed chemical compositions.

### 2.2. Catalytic Ability of the HEOs

The catalytic activities and the chemo-selectivities toward the different products formed during the pinacol-type oxidative coupling reaction of the aldehydes are presented using the HEOs as the actual catalysts. The physicochemical properties of the HEO catalysts are summarized in Table 2. It was found that all textural and structural characteristics of the different HEOs—such as the crystallite sizes and structural parameters—are very similar, while the specific surface areas (48–103 m^2^/g) and total pore volumes (0.08–0.33 cm^3^/g) are not very different. However, CZLPY has a significantly higher surface area than the other HEOs. Several authors [42,43,44,45,46] suggested that the addition of rare-earth cations affects the change in the specific surface area values. CZLPY consists of three cations (Zr, Pr, Y), which are responsible for an increase in its specific surface area, while all the others consist of two. Therefore, a much higher specific surface area of CZLPY could be the result of the synergistic effect of the involved cations. Moreover, significant differences could be observed in the acidities of HEOs, which increase in the order of CZLEY < CZLPG < CZEYG < CZLPY. Consequently, the effect of the acidity should be mainly studied when comparing the catalytic indicators of the different samples presented in Table 1.

**Scheme 1:**
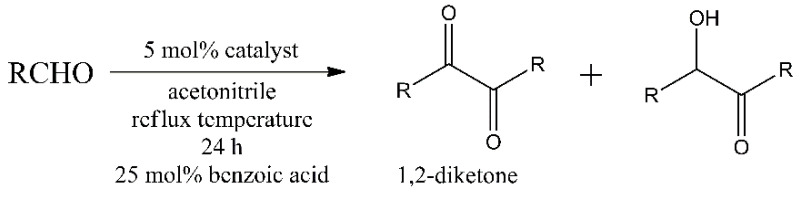
Pinacol-type oxidative coupling reaction of the aldehydes.

**Figure 4:**
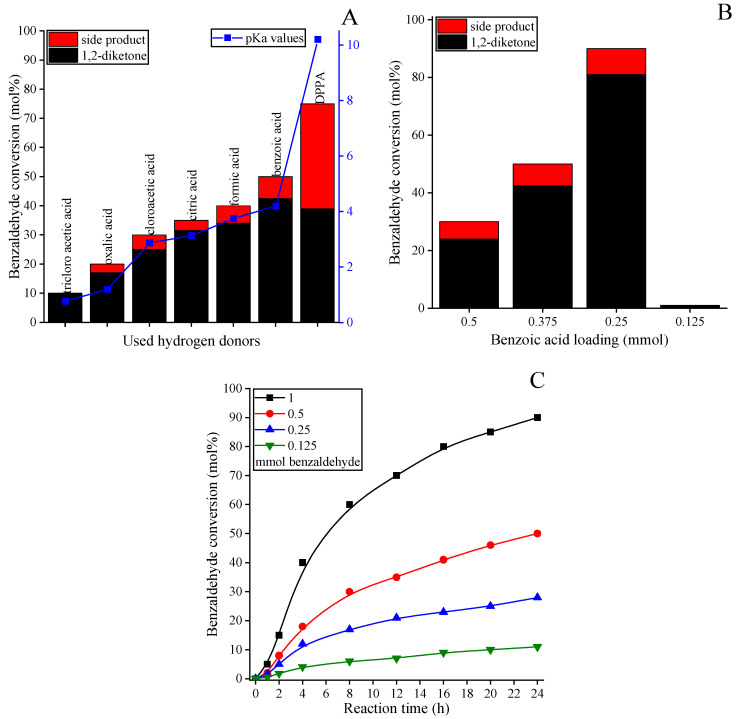
Benzaldehyde conversions as a function of the quality of the hydrogen donors (**A**) and benzoic acid loading (**B**). Time profiles of benzaldehyde conversions at different benzaldehyde loadings (**C**). Reaction conditions: 1 mmol benzaldehyde, 2 cm^3^ acetonitrile, 0.5/0.25 mmol organic acid/benzoic acid, 5 mol% CZLPY catalyst, reflux temperature, 24 h.

The oxidative pinacol-type couplings (Scheme 1) were performed in the presence of catalytic amounts of the HEOs (5 mol% for metal ions) by using diphenylphosphoryl azide (DPPA) or organic acids as the external acids at reflux temperature, in 1,2-dichloromethane, for 24 h. Our studies were initiated by probing reaction conditions to maximize the diketone yield upon converting benzaldehyde in the presence of CZLPY and DPPA. Although the desired transformation was slightly viable in the absence of HEO and external acid, respectively, CZLPY seemed effective, providing an acceptable diketone yield with minor diketone selectivity in the case of employing DPPA as the additive (Table S5). Lower reaction temperatures induced a significant decrease in the aldehyde conversion, as well as a notable decrease in the diketone selectivity; thus, the reflux temperature was used as follows (Tables S6 and S8). By screening the potential solvents for oxidative coupling, besides the dichloromethane, only the acetonitrile proved to be useful, achieving almost the same reaction rate as in the halogenated solvent (Table S7). The most relevant effects on the diketone yields and selectivities could be attributed to the quality and the quantity of the additive. By systematically altering the additive dropped, it could be easily assumed that the application of organic acids with higher pKa values led to a significant increase in the aldehyde conversions, however with a dramatic decrease in the diketone selectivities (Figure 4A). The use of benzoic acid seemed to be the best choice taking into account that a highly diketone-selective (~85%) reaction with average aldehyde conversion could be realized in the presence of it. Additionally, upon reducing the amount of benzoic acid added, higher reaction rates were able to be obtained as long as a limit value was obtained (Figure 4B). Under that value, the benzoic acid transformed totally into chalcone in the presence of the catalyst in such a way that the benzaldehyde remained unchanged. Finally, the catalyst loading was optimized. It was found that the use of catalyst portions other than 5 mol% led to significant erosion in either diketone selectivity (10 mol%) or aldehyde conversion (1.25–2.5 mol%) (Table S9). Nevertheless, up to 90% benzaldehyde conversion with 90% diketone selectivity could be accomplished under the optimized reaction conditions. These results are very competitive compared even to Pd-based homogeneous catalysts as well [30]. The kinetic profile of the oxidative coupling indicated that the initial reaction rate for the catalyst was more than one, which may indicate that the aldehyde activation is the rate-limiting step of the overall catalytic reaction (Figure 4C). This activation step may be related to the additive with a Brønsted acid character [47].

**Figure 5:**
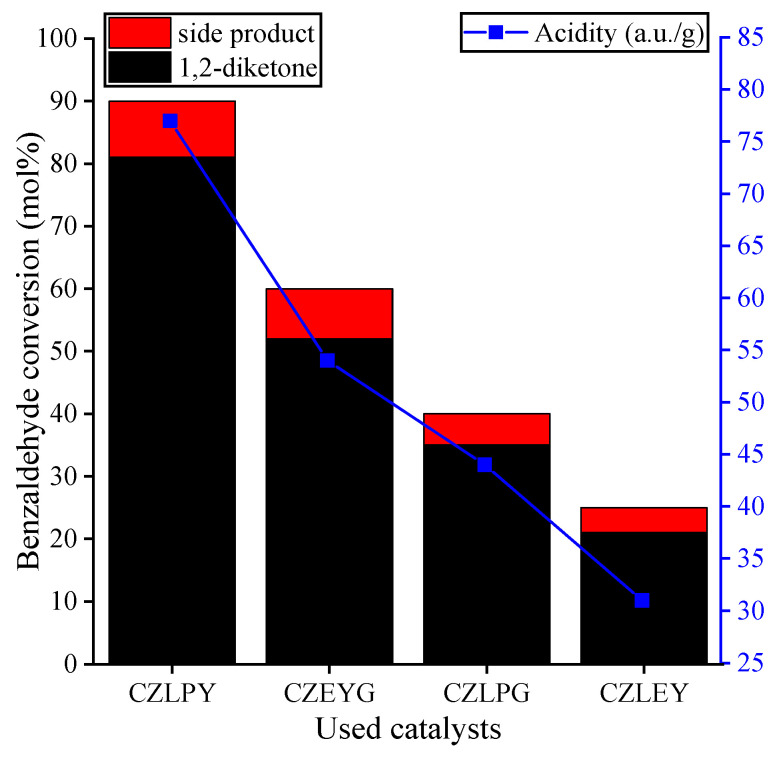
Comparative study of the catalytic ability of the different HEOs to promote the oxidative pinacol-type coupling reaction of the benzaldehyde. Reaction conditions: 1 mmol benzaldehyde, 2 cm^3^ acetonitrile, 0.25 mmol benzoic acid, 5 mol% catalyst, reflux temperature, 24 h.

With the optimized reaction conditions in hand, the catalytic abilities of the HEOs with different compositions and acidities were compared to each other. For a valid comparison, all the reactions were carried out keeping the ratio of the aldehyde-to-metal ion constant at 5 mol%. On the one hand, in all cases, the desired diketone was produced with almost the same selectivity (Figure 5). Consequently, the quality of the additive and its measure dictated the ratio of the desired product. On the other hand, CZLPY exhibited the highest activity of the series, and the order in the total activity followed the same trend as during the acidity measurements (Figure 5). A general trend in increasing aldehyde conversions with increasing acidity was observed. The remarkable activity of the HEOs, especially CZLPY, was reflected by carrying out oxidative coupling with a pure/single oxide of the framework cations and their physically mixed composites, all falling short of providing the desired product with comparable efficiency (Table S10). As can be seen in Table S10, Zr-containing probe catalysts proved to be more efficient compared to other ones. This fact enabled us to suppose that the Zr centers played the key role during the catalytic reactions, the activities of which were enhanced by synergetic effects established in the mixed oxide lattice. Though there is no concrete evidence of the reaction mechanism, similar to the work of Chen et al. [48], a cooperative interplay of Brønsted acid and Lewis acid sites, including dehydrogenation of the product of the coupling under Brønsted acid sites, is the most probable way, which could be related to this reaction. Because HEOs have more than one type of active Lewis acid dispersed uniformly, unlike a physically mixed composite, their activities were notably enhanced compared to the oxides used for the comparison.

**Figure 6:**
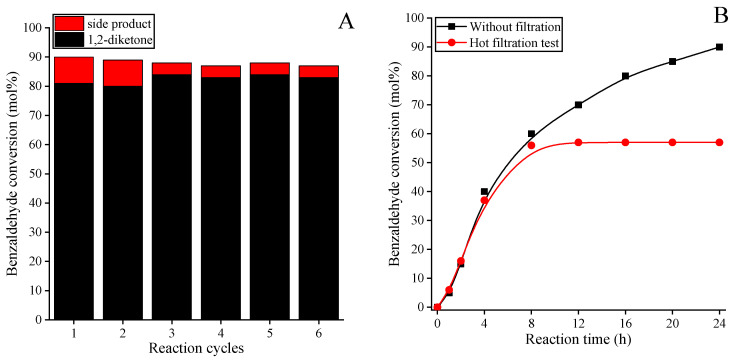
Recyclability test of the CZLPY catalyst (**A**). Heterogeneity (hot filtration) test of the pinacol-type oxidative coupling reaction of the benzaldehyde catalyzed by the CZLPY catalyst (**B**). Reaction conditions: 1 mmol benzaldehyde, 2 cm^3^ acetonitrile, 0.25 mmol benzoic acid, 5 mol% catalyst, reflux temperature, 24 h.

The possible deactivation of CZLPY and its reusability were studied by recycling the used HEO after each cycle followed by being intensively washed with acetonitrile, and the reaction was upscaled six times. It could be determined that the conversion was practically complete even after six reuses. In addition, surprisingly, higher selectivities (~95%) to the desired product were detected starting with the third cycle (Figure 6A). The Raman analysis performed on the used catalyst, after the washing step, revealed that no relevant gradual destruction of the HEO had occurred from one to six runs, except for a slight decrease in the intensity of the *F_2g_* and defect bands, as shown in Figure S4.

Next, to establish the heterogeneity of the reaction, a hot filtration test was carried out by separating the catalyst from the reaction mixture (filtrate) after 16 h (60% conversion). This filtration test made it clear that the reaction does not proceed further in the filtrate. This test exhibited that CZLPY mixed oxide can be handled as a heterogeneous catalyst (Figure 6B).

Subsequently, the scope of the multimetallic CZLPY proved to be good under otherwise identical reaction conditions (Table 3.). An appropriate substrate tolerance could be seen, exhibiting the versatility and flexibility of this multimetallic catalyst.

## 3. Materials and Methods

### 3.1. Materials

Commercially available chemicals were used for syntheses as follows: cerium(III) nitrate hexahydrate 99.99%, zirconium(IV) oxynitrate hydrate 99%, lanthanum(III) nitrate hexahydrate 99.99%, praseodymium(III) nitrate hexahydrate 99.9%, europium(III) nitrate hexahydrate 99.9%, gadolinium(III) nitrate hexahydrate 99.9% from Sigma Aldrich, Germany; yttrium(III) nitrate hexahydrate 99.9% from Alfa Aesar, Germany; concentrated ammonia solution 25% from Gram-Mol, Croatia; citric acid monohydrate 99.9% from T.T.T., Croatia. To perform the catalytic reactions the analytical grade products acetaldehyde (97%), propionaldehyde (97%), butyraldehyde (97%), benzaldehyde (97%), furfural (97%), vanillin (94%), acetonitrile (99%), benzoic acid (98%), 1,2-dichloromethane (99%), 2-methylhydrofuran (99%), heptane (99%), dimethyl sulfoxide (DMSO) (99%), ethyl-acetate (EtOAc) (99%), and EtOH (96%) from Sigma-Aldrich were applied without further purification.

### 3.2. Synthesis of the HEOs

**Scheme 2:**
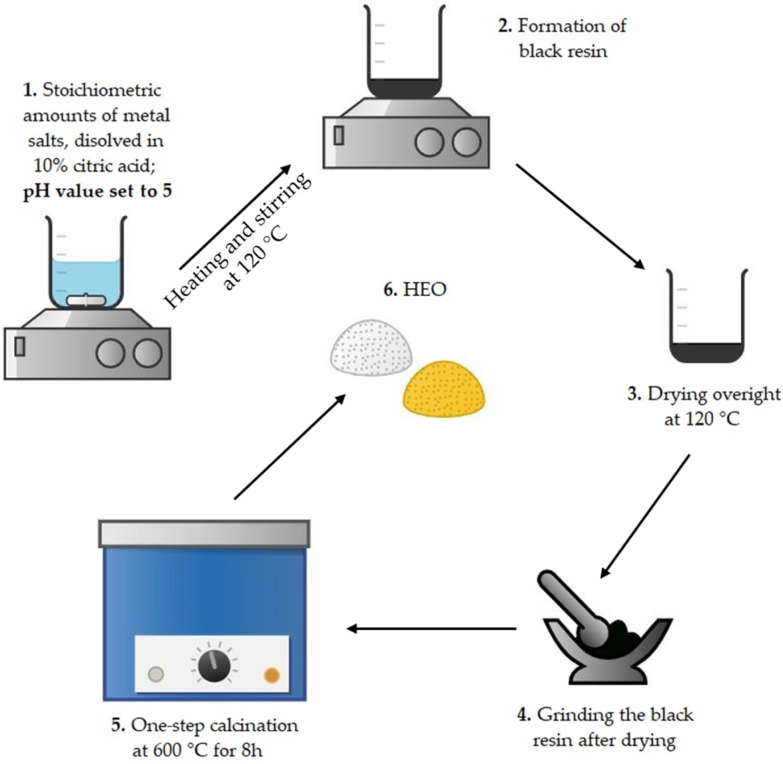
Schematic overview of the synthetic procedure.

The high-entropy oxides were synthesized using a modified, aqueous sol-gel citrate route [49,50] previously reported as a successful route for metal oxides’ synthesis. The synthetic procedure is shown in Scheme 2 below. Metal precursors were dissolved in their nitrate forms in a previously prepared 10% aqueous solution of citric acid (10 g of citric acid in 100 mL of MiliQ water) in a total amount of 1 mmol, following the targeted formula (Ce_0.2_Zr_0.2_C_0.2_D_0.2_E_0.2_O_2_, where *C-E* are different rare-earth metal cations and O is oxygen). The solution with dissolved metal precursors was stirred on a magnetic stirrer (IKA C-MAG HS 7, Staufen, Germany) for 30 min before adjusting pH value to 5 using concentrated ammonia solution (pH-meter 211, HANNA, Zagreb, Croatia). The reaction mixture was then treated with constant mixing on a magnetic stirrer, followed by heating at 120° until the water evaporated and black resin formed. The black resin was further dried overnight at 120 °C in a drying oven (Instrumentaria ST-01/02, Sesvete, Croatia) to evaporate excess water. After that, it was ground with a pestle in a mortar, followed by one-step calcination (Furnace SN 342689, Nabertherm GmbH, Lilienthal, Germany) at 600 °C for 8 h, with a heating rate of 4 °C/min.

### 3.3. Methods

#### 3.3.1. Powder X-ray Diffraction

Powder X-ray diffraction patterns were collected on a PANalytical Aeris Research Diffractometer in θ-θ geometry (Malvern PANalytical, Malvern, UK), using CuKα radiation (40 kV, 15 mA) at 298 K. The range of 2θ was from 20°–100°, with a step size of 0.02. Rietveld refinement was provided by the computer program FULLPROF [51]. The crystal structures of the synthesized compounds were visualized using the VESTA software [52].

#### 3.3.2. Brunauer–Emmett–Teller Surface Area Measurement

Pore volumes and surface areas were investigated by Autosorb iQ-MP (Quantachrome, Boynton Beach, FL, USA), using multipoint BET. All compounds were previously degassed at 200 °C for 2 h.

#### 3.3.3. Raman Spectroscopy

Raman spectra were recorded with a Raman Senterra II (Bruker, Billerica, MA, USA) microscope at an excitation wavelength of 765 nm applying a 12.5 mW laser power and averaging 20 spectra with an exposition time of 20 s.

#### 3.3.4. SEM, TEM, and STEM-EDX Measurements

A scanning electron microscope (Thermo Fisher Scientific Apreo C, Thermo Fisher Scientific, Waltham, MA, USA ) was employed to examine the surface structure. TEM and STEM-EDX data were collected using a double-aberration-corrected Themis-Z microscope (Thermo Fisher Scientific, Waltham, MA, USA ) at an accelerating voltage of 300 kV, which was equipped with a Oneview IS camera (Ametek) and a Super-X EDX detector (Thermo Fisher Scientific). The powder sample was dispersed on a lacey carbon-coated gold grid and loaded onto a TEM holder for measurements. For the STEM-EDX maps, the probe current was about 0.2 nA with a dwell time of 10 μs. The EDX data were prepared using the Velox (Thermo Fisher Scientific, Version 2.12.1.37) software.

#### 3.3.5. ICP-MS Measurements

An inductively coupled plasma mass spectrometer, Agilent 7900 ICP-MS (Agilent Technologies, Singapore, Singapore), was used for all measurements. The instrument was equipped with standard nickel sampling and skimmer cones, a standard glass concentric nebulizer, a quartz spray chamber, and a quartz torch with a 2.5 mm ID injector. All samples were analyzed as two replicates. A certified reference material of rare-earth ore was used to test the accuracy of the method.

#### 3.3.6. Temperature-Programmed Desorption of NH_3_

The temperature-programmed desorption (TPD) was performed in a BELCAT-A apparatus (BEL Japan, Inc., Osaka, Japan) using a reactor (quartz tube with a 9 mm outer diameter) that was externally heated. Before the measurements, the catalyst samples were pretreated at 473 K. Then, the sample was cooled in flowing He to 323 K and equilibrated for 15 min. The samples were flushed with NH_3_ for 30 min and then flushed with He for 15 min at 323 K. The reactor was heated at a heating rate of 10 K·min^−1^ up to 800 K. The amount of the ammonia was detected by a thermal conductivity detector (TCD).

### 3.4. Catalytic Reaction Procedures

#### 3.4.1. Pinacol-Type Oxidative Coupling of the Aldehydes

The optimized procedure for the catalytic pinacol-type oxidative coupling of the aldehyde was as follows. Acetonitrile (2 cm^3^), the corresponding aldehyde (1.0 mmol, 1.0 equiv.), benzoic acid (0.25 mmol, 0.25 equiv.), and the corresponding HEO as the catalyst (corresponding to a 5 mol% metal ion loading) were combined in a nitrogen-flushed Schlenk tube equipped with a magnetic stir bar. The reaction mixture was stirred at reflux temperature for 24 h. Then, the mixture was cooled to room temperature, and the resultant liquid was extracted with brine (3 × 15 mL). The organic layer was dried over Na_2_SO_4_ and concentrated under reduced pressure. Not only the activity, but also the reusability of the potential catalyst was investigated in the heterocyclization reaction. The conversion and selectivity were determined after each reaction by GC-MS using a Thermo Scientific Trace 1310 Gas Chromatograph (Thermo Fisher Scientific, Waltham, MA, USA) coupled to a Thermo Scientific ISQ QD Single Quadrupole Mass Spectrometer (Thermo Fisher Scientific, Waltham, MA, USA) using a Thermo Scientific TG-SQC column (15 m × 0.25 mm ID × 0.25 μm film) (Thermo Fisher Scientific, Waltham, MA, USA). During the measurements, the parameters were as follows: column oven temperature: from 50 °C to 300 °C at 15 °C min^−1^; injection temperature: 240 °C; ion source temperature: 200 °C; electrospray ionization: 70 eV; carrier gas: He at 1.5 mL min^−1^; injection volume: 2 μL; split ratio: 1 to 33.3; mass range: 25–500 *m*/*z*. Starting materials, products, and byproducts were identified using reference samples.

#### 3.4.2. Hot Filtration Test

The coupling reaction was carried out under the optimized reaction conditions. The bulk catalyst was readily removed by a simple filtration after 12 h followed by the treatment of the filtrate under the optimized reaction conditions for another 12 h.

## 4. Conclusions

Four ceria–zirconia-based high-entropy catalysts were successfully synthesized. The applied synthetic route, the modified sol-gel citrate route, resulted in phase-pure compounds with a cubic structure, with lattice parameters that differ from pure CeO_2_. This is related to the lattice expansion/contraction due to the incorporation of five cations into a single-cation lattice. The investigation of the physicochemical properties of the newly developed and synthesized catalysts shows that the crystallite size, lattice parameters, surface areas, and pore volumes are similar, while the Lewis acidity differs significantly.

The pinacol-type oxidative coupling reaction of the aldehydes was presented, using HEOs as the actual catalysts, which demonstrated the catalytic abilities and chemoselectivity of the catalysts. The reaction conditions were optimized followed by a comparative study of the HEOs under the same conditions. Upon using the HEOs as the catalysts, the desired diketone product was produced with almost the same selectivity, unlike the activity, which followed the trend of increasing acidity. The remarkable activity of the HEOs was proven in comparison with the pure building block oxides and their physically mixed composites, especially in the case of the CZLPY compound.

CZLPY oxide proved to be a versatile, reusable, and heterogeneous catalyst, taking into account the results of the recycling and hot filtration test, as well as the scope.

## Figures and Tables

**Table 1:** Synthesized HEOs with their corresponding chemical formulae.

Compound	Chemical Formula
CZLEY	Ce_0.2_Zr_0.2_La_0.2_Eu_0.2_Y_0.2_O_2_
CZLPY	Ce_0.2_Zr_0.2_La_0.2_Pr_0.2_Y_0.2_O_2_
CZEYG	Ce_0.2_Zr_0.2_Eu_0.2_Y_0.2_Gd_0.2_O_2_
CZLPG	Ce_0.2_Zr_0.2_La_0.2_Pr_0.2_Gd_0.2_O_2_

**Table 2:** Physicochemical characteristics of the investigated compounds.

Compound	Pore Volume (cm^3^/g)	S_BET_ (m^2^/g)	Average Crystallite Size (nm)	Acidity (a.u./g)
CZLEY	0.14	48	6	31
CZLPY	0.27	103	6	77
CZEYG	0.08	51	5	54
CZLPG	0.33	60	6	44

**Table 3:** Pinacol-type oxidative coupling reactions of the different aldehydes promoted by the CZLPY catalyst. 1 mmol aldehyde, 2 cm^3^ acetonitrile, 0.25 mmol benzoic acid, 5 mol% catalyst, reflux temperature, 24 h.

Aldehydes	Products	Aldehyde Conversion (mol%)	Product Yield (mol%)
Acetaldehyde	Diacetyl	80	70
Propionaldehyde	3,4-hexanedione	76	68
Butyraldehyde	4,5-octanedione	71	61
Benzaldehyde	Benzil	90	81
Furfural	Furil	73	66
Vanillin	1,2-bis-benzo(1,3)diioxol-5-yl-ethane-1,2-dione	67	60

## Data Availability

The data presented in this study are available on request from the corresponding author.

## Supplementary Materials

The following are available online. Table S1: Rietveld refinement parameters and crystallographic data obtained from the PXRD pattern, Table S2: Relative oxygen vacancies’ concentration, calculated from Raman spectra, Figure S1: SEM images of: CZLEY (a); CZLPY (b); CZLPG (c); CZEYG (d), Figure S2: Qualitative EDX elemental maps of the powder CZLPY sample, Table S3: Quantitative results of the EDX spectrum of CZLPY, Figure S3: HAADF image and EDX spectrum of CZLPY, Table S4: ICP-MS results of the elemental analysis of all four compounds, Table S5: Benzaldehyde’s direct conversion to benzil. Reaction conditions: 1 mmol benzaldehyde, 2 cm^3^ acetonitrile, 0.5 mmol DPPA, 5 mol% CZLPY catalyst, reflux temperature, 24 h, Table S6: Benzaldehyde direct conversion to benzil. Screening of the reaction temperature. Reaction conditions: 1 mmol benzaldehyde, 2 cm^3^ acetonitrile, 0.5 mmol DPPA, 5 mol% CZLPY catalyst, 24 h, Table S7: Benzaldehyde’s direct conversion to benzil. Screening of the applied solvent. Reaction conditions: 1 mmol benzaldehyde, 2 cm^3^ solvent, 0.5 mmol DPPA, 5 mol% CZLPY catalyst, reflux temperature, 24 h, Table S8: Benzaldehyde’s direct conversion to benzil. Screening of the reaction temperature (in acetonitrile). Reaction conditions: 1 mmol benzaldehyde, 2 cm^3^ acetonitrile, 0.5 mmol DPPA, 5 mol% CZLPY catalyst, 24 h, Table S9: Benzaldehyde’s direct conversion to benzil. Screening of the catalyst loading. Reaction conditions: 1 mmol benzaldehyde, 2 cm^3^ acetonitrile, 0.25 mmol benzoic acid, CZLPY catalyst, reflux temperature, 24 h, Table S10: Benzaldehyde’s direct conversion to benzil. Screening of the applied catalyst. Reaction conditions: 1 mmol benzaldehyde, 2 cm^3^ acetonitrile, 0.25 mmol benzoic acid, 5 mol% catalyst, reflux temperature, 24 h, Figure S4: Comparison of the Raman spectra of the CZLPY catalyst as synthesized and after the washing step.

## Sample Availability

Samples of the compounds are available from authors.
