# Supplementary material for: Sol-Gel Synthesis of Ceria-Zirconia-Based High-Entropy Oxides as High-Promotion Catalysts for the Synthesis of 1,2-Diketones from Aldehyde
Source: Molecules. 2021 Oct 10;26(20):6115. doi: 10.3390/molecules26206115 (PMC8539213; doi:10.3390/molecules26206115)
Supplement: Supplementary file 1 [file molecules-26-06115-s001.zip › molecules-1388250-supplementary.pdf]

Sol-gel Synthesis of Ceria-Zirconia Based High Entropy Oxides as High Promotion Catalysts for the Synthesis of 1,2-Diketones from Aldehyde

Dalibor Tatar [1], Jelena Kojčinović [1], Berislav Marković [1], Aleksandar Széchenyi [1], Aleksandar Miletić [2], Sándor Balázs Nagy [3], Szilveszter Ziegenheim [3], Imre Szenti [4], Andras Sapi [4], Ákos Kukovecz [4], Kristijan Dinjar [5], Yushu Tang [6], David Stenzel [6], Gábor Varga [7,*], and Igor Djerdj [1,*]

[1] Department of Chemistry, Josip Juraj Strossmayer University of Osijek, Cara Hadrijana 8/A, HR-31000 Osijek, Croatia; dtatar@kemija.unios.hr (D. T.), jbijelic@kemija.unios.hr (J. K.), bmarkovi@kemija.unios.hr (B. M.), szealex@kemija.unios.hr (A. Sz.)

[2] Faculty of Technical Sciences, University of Novi Sad, Trg Dositeja Obradovića 6, SRB-21000 Novi Sad, Serbia; aleksandarvmiletic@gmail.com (A. M.)

[3] Department of Organic Chemistry, University of Szeged, Dóm tér 8., H-6720 Szeged, Hungary; sandorb@chem.u-szeged.hu (S. B. N.), ziegenheimsz@chem.u-szeged.hu (Sz. Z.)

[4] Department of Applied and Environmental Chemistry, University of Szeged, Rerrich Béla sq. 1., H-6720 Szeged, Hungary; sapia@chem.u-szeged.hu (A. S.), kakos@chem.u-szeged.hu (Á. K.), szentiimre@gmail.com (I. S.)

[5] Department of Otorhinolaryngology and Maxillofacial Surgery, Faculty of Medicine, Josip Juraj Strossmayer University of Osijek, Cara Hadrijana 10/E, HR-31000 Osijek, Croatia; kristijan.dinjar@kbco.hr (K. D.)

[6] Karlsruhe Institute of Technology (KIT), Institute of Nanotechnology, Hermann-von-Helmholtz-Platz 1, DE-76344 Eggenstein-Leopoldshafen, Germany; yushu.tang@kit.edu (Y. T.), david.stenzel@kit.edu (D. S.)

[7] Department of Physical Chemistry and Materials Science, University of Szeged, Rerrich Béla sq. 1., H-6720 Szeged, Hungary

* Correspondence: gabor.varga5@chem.u-szeged.hu (G. V.), igor.djerdj@kemija.unios.hr (I. Dj.); Tel.: +36-62-343-795 (G. V.), +385-31-399-975 (I. Dj.)

Table S1. Rietveld refinement parameters and crystallographic data obtained from the PXRD pattern.

Compound	CZLEY	CZLPY	CZEYG	CZLPG
Space Group		*Fm-3m*		
Crystal system		Cubic		
Data collection range		20 – 100°		
Phase composition (wt %)		100		
Molecular weight	138.23	136.01	141.91	149.68
Z	8	8	8	8
Lattice parameters (Å)	a = 5.4558(2)	a = 5.4517(2)	a = 5.4583(2)	a = 5.4601(2)
Cell volume (Å^3)	162.394(6)	162.035(6)	162.616(6)	162.777(6)
Calculated density (g/cm^3)	6.624	6.235	6.582	6.938
No. of parameters refined	16	15	16	16
No. of bond lengths restrained	0	0	0	0
No. of bond angles restrained	0	0	0	0
Average apparent crystallite size (nm)	6	6	5	6
Average apparent microstrains (×10^{-4})	41.055	25.221	50.302	34.438
R_B (%)	1.06	1.37	1.01	1.35
R_p (%)	12.6	11.0	13.5	13.0
R_{wp} (%)	9.12	8.79	9.44	9.53
R_e (%)	8.62	8.37	10.0	10.3
χ^2	1.12	1.103	0.888	0.849

Table S2. Relative oxygen vacancies concentration in HEOs, calculated from Raman spectra.

Compound	Integral intensities ratio (II_D/II_{F2g})
CZLEY	1.30
CZLPY	1.81
CZEYG	1.44
CZLPG	1.90

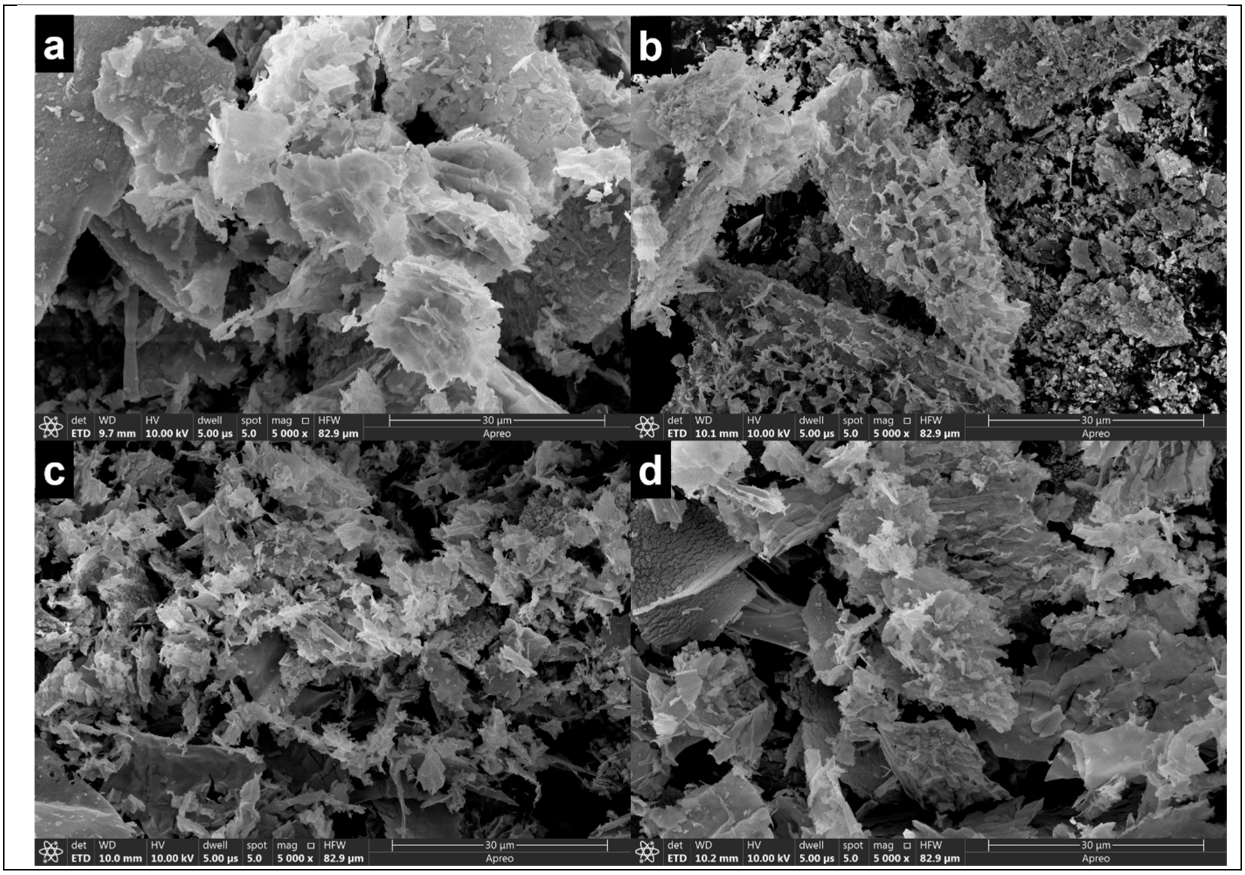

Figure S1. SEM images of: CZLEY (a), CZLPY (b), CZLPG (c), CZEYG (d).

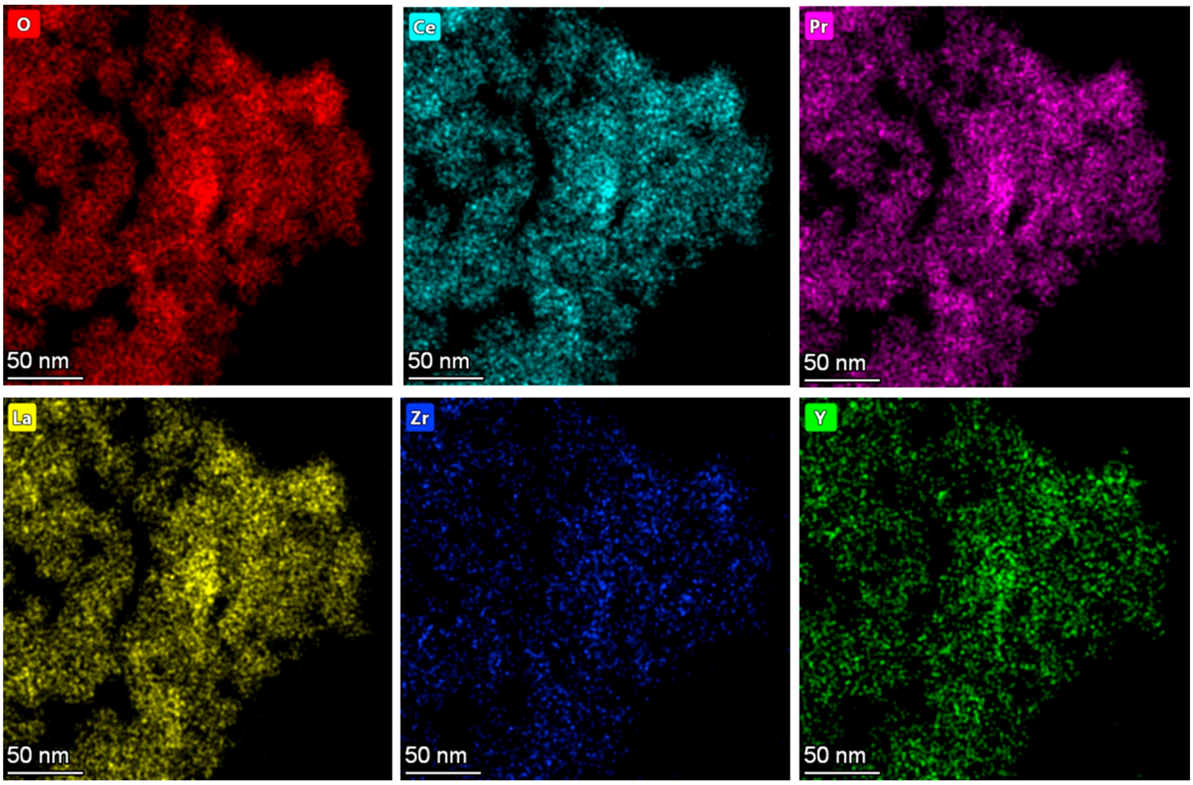

Figure S2. Qualitative EDX elemental maps of powder CZLPY sample.

Table S3. Quantitative results of EDX spectrum of CZLPY.[a]

Z	M(g/mol)	Element	Family	Atomic Fraction (%)	Nominal Atomic Fraction (%)	Mass Fraction (%)
8	16	**O**	K	69.01	66.65	22.55
39	88.91	**Y**	K	6.05	6.67	10.99
40	91.22	**Zr**	K	4.82	6.67	8.98
57	138.9	**La**	L	6.99	6.67	19.82
58	140.1	**Ce**	L	6.16	6.67	17.64
59	140.9	**Pr**	L	6.96	6.67	20.03

[a] Standard deviation: <5% of atomic concentration.

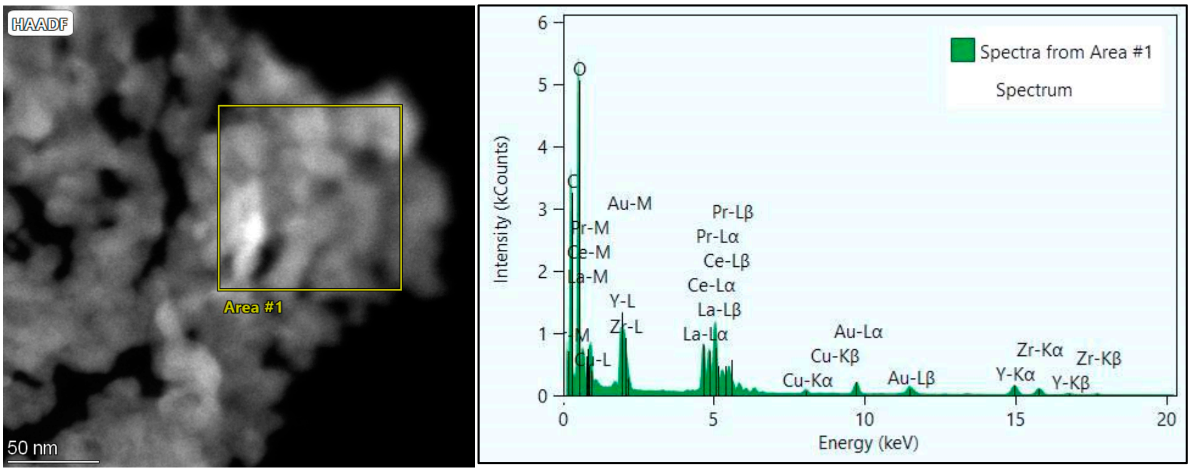

Figure S3. HAADF image and EDX spectrum of CZLPY.

Table S4. Results of ICP-MS analysis (mass fractions) of synthesized compounds compared to nominal (theoretical) values. [a]

Compound (nominal composition)	Element	w (theoretical) / %	w (measured) / %
$Ce_{0.2}Zr_{0.2}La_{0.2}Eu_{0.2}Y_{0.2}O_2$	Ce	18,17	15,52
	Zr	11,83	9,61
	La	18,01	18,61
	Eu	19,71	19,74
	Y	11,53	11,13
$Ce_{0.2}Zr_{0.2}La_{0.2}Pr_{0.2}Y_{0.2}O_2$	Ce	18,43	15,43
	Zr	12,00	11,73
	La	18,28	18,81
	Pr	18,54	19,22
	Y	11,70	11,69
$Ce_{0.2}Zr_{0.2}Eu_{0.2}Y_{0.2}Gd_{0.2}O_2$	Ce	17,75	20,04
	Zr	11,56	11,08
	Eu	19,25	17,97
	Y	11,26	11,87
	Gd	19,92	16,68
$Ce_{0.2}Zr_{0.2}La_{0.2}Pr_{0.2}Gd_{0.2}O_2$	Ce	16,91	20,21
	Zr	11,01	11,62
	La	16,76	19,96
	Pr	17,00	19,88
	Gd	18,98	21,62

[a] The relative standard deviation is 10-30%

Table S5. Benzaldehyde direct conversion to benzil. Reaction conditions: 1 mmol benzaldehyde, 2cm^3 acetonitrile, 0.5 mmol DPPA, 5 mol% CZLPY catalyst, reflux temperature, 24 h.

Catalysts	Additive	Benzaldehyde conversion (mol%)	Benzil selectivity (mol%)
–	–	–	–
CZLPY	–	11	30
–	DPPA	10	20
CZLPY	*DPPA*	*80*	*49*

Table S6. Benzaldehyde direct conversion to benzil. Screening of the reaction temperature. Reaction conditions: 1 mmol benzaldehyde, 2cm^3 acetonitrile, 0.5 mmol DPPA, 5 mol% CZLPY catalyst, 24 h.

**Reaction temperature (°C)**	**Benzaldehyde conversion (mol%)**	**Benzil selectivity (mol%)**
10	15	16
20	35	15
30	55	20
Reflux	*80*	*49*

Table S7. Benzaldehyde direct conversion to benzil. Screening of the applied solvent. Reaction conditions: 1 mmol benzaldehyde, 2cm^3 solvent, 0.5 mmol DPPA, 5 mol% CZLPY catalyst, reflux temperature, 24 h.

**Solvents**	**Benzaldehyde conversion (mol%)**	**Benzil selectivity (mol%)**
1,2-dichloromethane	80	49
DMSO	12	40
DMF	—	—
Acetonitrile	*75*	*52*
Heptane	—	—
EtOAc	4	60
EtOH	1	70
EtOAc : EtOH 3:1	15	100

Table S8. Benzaldehyde direct conversion to benzil. Screening of the reaction temperature (in acetonitrile). Reaction conditions: 1 mmol benzaldehyde, 2cm^3 acetonitrile, 0.5 mmol DPPA, 5 mol% CZLPY catalyst, 24 h.

**Reaction temperature (°C)**	**Benzaldehyde conversion (mol%)**	**Benzil selectivity (mol%)**
25	20	15
40	35	18
55	59	19
Reflux	*75*	*52*

Table S9. Benzaldehyde direct conversion to benzil. Screening of the catalyst loading. Reaction conditions: 1 mmol benzaldehyde, 2cm[3] acetonitrile, 0.25 mmol benzoic acid, CZLPY catalyst, reflux temperature, 24 h.

**Catalyst loading (mol%)**	**Benzaldehyde conversion (mol%)**	**Benzil selectivity (mol%)**
1.25	30	91
2.5	50	88
5	*90*	*90*
10	93	61

Table S10. Benzaldehyde direct conversion to benzil. Screening of the applied catalyst. Reaction conditions: 1 mmol benzaldehyde, 2cm^3 acetonitrile, 0.25 mmol benzoic acid, 5 mol% catalyst, reflux temperature, 24 h.

**Catalysts**	**Benzaldehyde conversion (mol%)**	**Benzil selectivity (mol%)**
CZLPY	*90*	*90*
CZEYG	60	87
CZLPG	40	88
CZLEY	25	84
CeO_2	23	85
La_2O_3	20	86
ZrO_2	41	95
Eu_2O_3	—	—
Y_2O_3	—	—
CeO_2:La_2O_3 1:1	24	84
CeO_2:ZrO_2 1:1	48	86
ZrO_2:La_2O_3 1:1	43	88
CeO_2:La_2O_3:ZrO_2 1:1:1	45	89

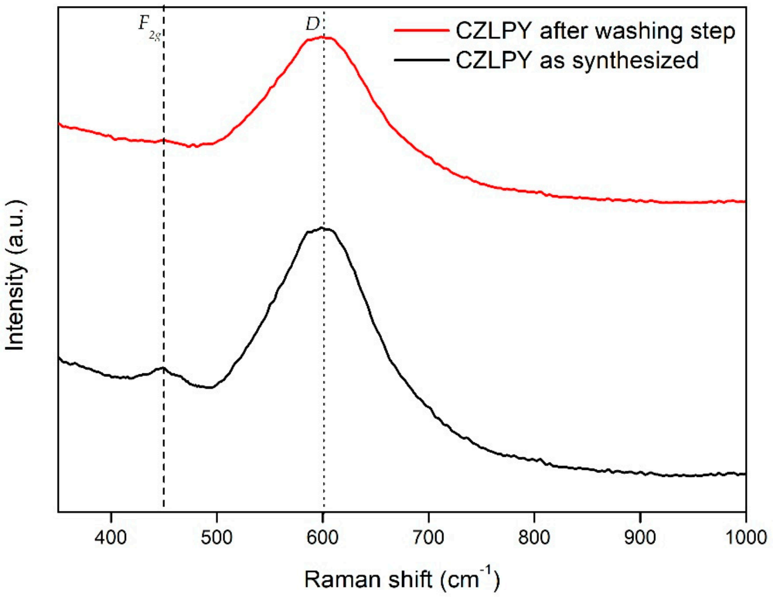

Figure S4. Compared Raman spectra of CZLPY catalyst as synthesized and after washing step.
